# Influence of Capsaicin Supplementation on the Enhancement of Passive Immunity Transfer Through Modulation of Immunoglobulin Absorption in Neonatal Calves

**DOI:** 10.3390/ani15121676

**Published:** 2025-06-06

**Authors:** Ermes R. Rodas, Luis E. Ayala, Jorge B. Dután, Gissela E. Gañan, José L. Pesántez, Juan V. González-Martín

**Affiliations:** 1 Faculty of Agriculture Sciences, University of Cuenca, Cuenca 010107, Ecuador; luis.ayala@ucuenca.edu.ec (L.E.A.); jorgeb.dutans@ucuenca.edu.ec (J.B.D.); gissela.gananc@ucuenca.edu.ec (G.E.G.); jose.pesantez@ucuenca.edu.ec (J.L.P.); 2 Department of Animal Medicine and Surgery, Faculty of Veterinary Medicine, Complutense University of Madrid (UCM), Avda Pta. de Hierro s/n, 28040 Madrid, Spain

**Keywords:** capsaicin, immunoglobulins, metabolites, enzymes, physiological parameters, bovines

## Abstract

In cattle, the transfer of passive immunity from mother to calf across the placenta is limited. Therefore, providing quality colostrum or supplementing with additives that enhance this attribute is crucial to optimise the transfer of passive immunity (TPI). The objective of the present study was to investigate the effect of capsaicin on the absorption of immunoglobulins, metabolites, and enzymes to optimise TPI in neonatal calves, and to assess its impact on selected physiological parameters. Two experimental groups were established: a control group (CON; *n* = 8), which received colostrum in three feedings (at 1, 12, and 20 h after calving), and a capsaicin group (CAP; *n* = 8) which received 40 mg of capsaicin per kilogram of body weight added to the colostrum. Birth weight (CON = 36.7 ± 1.10 kg; CAP = 36.2 ± 2.64 kg) and weight at 48 h of age (CON = 38.5 ± 1.11 kg; CAP = 38.0 ± 2.82 kg) were similar between the two experimental groups. At 48 h post-colostrum feeding, the CAP group exhibited significantly higher serum concentrations of IgG, total protein, and albumin—up to 23.4 times greater than those observed in the CON group (*p* < 0.001). Moreover, levels of glucose, cholesterol, and triglycerides were significantly elevated in the CAP group compared to the CON group (*p* < 0.05). Similarly, higher concentrations of alkaline phosphatase, aspartate aminotransferase, and alanine aminotransferase were observed in the CAP group. These findings suggest that the inclusion of 40 mg of capsaicin per kilogram of body weight in colostrum is safe and contributes positively to improving TPI in crossbred Holstein calves raised at altitudes higher than 2500 m above sea level.

## Simple Summary

It is characteristic of cattle that the transfer of maternal immunity to the calf occurs passively through colostrum. Therefore, any action that improves this transfer will improve resistance to infections and, consequently, the health of the calf, resulting in a reduction in antibiotic use. The study evaluated the effects of colostrum supplementation with 40 mg of capsaicin/kg of body weight in Holstein-crossbred calves raised in regions above 2500 m above sea level, with special emphasis on its impact on passive immune transfer (TPI). The results demonstrated that capsaicin supplementation improves serum concentrations of immunoglobulin G (IgG), total protein (TP), and albumin 48 h after calving. Increased availability of key metabolites and enzymes was also observed, which could reduce morbidity and mortality in calves. These findings support the development of neonatal management strategies aimed at optimising colostrum administration to mitigate the detrimental effects of failure of passive transfer of immunity (FTPI), a major concern in livestock production systems.

## 1. Introduction

The profitability of a dairy enterprise largely depends on the rate of live-born calves successfully reared to puberty [1]. Perinatal mortality ranges from 2.4% to 9.7%, and despite substantial advances in animal breeding, its prevalence remains high, particularly in Holstein dairy farms [2]. Various factors related to the rearing stage are being studied in an effort to reduce this gap.

One of these factors is the transfer of passive immunity (TPI) via colostrum, essential for the survival and health of neonatal calves [3]. Several factors can influence the efficacy of TPI, including the immunoglobulin (Ig) concentration of the colostrum [4], the timing of colostrum harvesting postpartum [5,6,7,8,9], the interval between parturition and first ingestion [10,11,12], the volume administered, the method of administration, and bacterial contamination [13]; all of these factors may contribute to the failure of passive immunity transfer (FTPI).

FTPI occurs when the neonate fails to absorb an adequate quantity of immunoglobulins [14]. This condition has been associated not only with increased morbidity and mortality, but also with lower weaning weights, reduced growth rates, and delayed onset of puberty [15,16]. When combined with poor hygiene, FTPI increases susceptibility to infectious diseases [17].

Colostrum is a rich source of nutrients and immunoglobulins, with IgG concentration being the primary indicator of its quality [7]. This quality can be assessed directly by measuring IgG concentration or indirectly using refractometry through Brix values. A reading above 21% corresponds to high-quality colostrum, equivalent to more than 50 mg/mL of IgG, whereas values below 20% indicate low-quality colostrum, containing less than 30 mg/mL of IgG [7,18].

Proper colostrum storage is essential for preserving quality and facilitating handling [19]. It can be refrigerated with or without preservatives [20] or frozen to prevent spoilage and bacterial proliferation [21]. Inadequate storage, however, may degrade its quality, affecting IgG content, energy, and protein levels [22].

Thawing is equally important to maintain colostrum integrity [22]. Pasteurisation is another widely used method to reduce or eliminate bacterial pathogens [23]. pasteurised colostrum can be stored under refrigeration for 8 to 10 days and has been shown to improve immunoglobulin absorption [24].

The prevalence of FTPI has significantly decreased in recent decades. For instance, in the United States it declined from 41% in a study conducted between 1991 and 1992 [25] to 19.2% in 2007 [26] and further to 12.1% in 2014 [27]. Nevertheless, high prevalence rates are still reported globally—for example, 41% in Italy [15], 24.8% to 33% in New Zealand [28], and between 21% and 43.3% in Canada [29].

Beyond improvements in dam and calf management, several strategies have been explored to enhance intestinal IgG absorption. These include colostrum supplementation with exogenous IgG or with additives that promote immune function [30] such as zeolites [31,32], selenium [33], and trypsin inhibitors [34,35].

Capsaicin, the active component responsible for the pungency of chilli peppers (*Capsicum annuum),* exhibits anti-inflammatory, antibacterial, and antioxidant properties. It also stimulates small intestinal development [36]. Although its role in animal nutrition is not yet fully elucidated, capsaicin has been suggested to enhance immune responses, stimulate digestive enzyme activity, and modulate metabolic profiles in livestock [36]. In one study, capsaicin administration in calves significantly increased serum concentrations of IgG, IgA, and IgM in a dose-dependent manner [36].

However, the mechanism by which capsaicin supplementation enhances immunoglobulin G (IgG) absorption has not yet been fully elucidated. Nevertheless, previous studies have shown that capsaicin significantly affects the expression of transporter proteins, particularly those involved in drug transport and in the regulation of the transcellular pathway across enterocytes, which may facilitate the non-selective absorption of macromolecules [37].

In addition, capsaicin also inhibits bacterial proliferation in the rumen and intestines of ruminants [38]; its antimicrobial activity is dose-dependent, attributed to the suppression of genes involved in bacterial development [39]. This effect is mediated through interaction with the transient receptor potential vanilloid 1 (TRPV1), expressed in nociceptive neurons and certain immune cells [40,41]. Previous studies have shown that capsaicin improves gastrointestinal health and reduces the proliferation of pathogenic bacteria in calves [36]. Additionally, Su et al. [36] reported that a daily dose of 0.3 mL of capsaicin enhanced the immune and antioxidant capacity of calves, improving disease tolerance and reducing inflammatory responses.

However, since capsaicin is an irritating compound, the dose and frequency of administration must be specific to each species, since its use has been associated with transient increases in body temperature, heart rate (HR), and respiratory rate (RR), this being due to the activation of the hypothalamic–pituitary–adrenal axis, resulting in the production of cortisol, a hormone that among its actions causes a temporary elevation of HR [42]. Nevertheless, it has been shown that the repetitive and prolonged use of capsaicin causes hyposensitivity in the TRV1 receptor, which prevents painful stimuli from releasing inflammatory neuropeptides in the synaptic space and, as a result, inhibits the transmission of painful stimuli and the sensation of pain to the spinal cord; therefore, it is considered that this may vary between species, so it is necessary to carry out specific studies [43].

Additionally, hypoxia that occurs at high altitudes (2500 m above sea level) is another factor that can alter physiological constants, increasing HR by 65%, hematocrit by 12%, and rectal temperature by 0.4 °C. Therefore, the values of physiological constants collected in our trial would not necessarily be linked solely to the addition of capsaicin [44]. So, it is necessary to deepen the knowledge about this product at this level of altitude.

However, as an irritant compound, capsaicin dosage and administration frequency must be species-specific, as its use has been associated with transient increases in body temperature, heart rate (HR), and respiratory rate (RR).

Based on these findings, it was hypothesised that the addition of capsaicin to colostrum could enhance TPI in neonatal calves. To test this, the concentrations of IgG and total protein (TP) were evaluated following the administration of supplemented colostrum. Additionally, the effects of capsaicin on serum albumin, selected metabolites, and enzyme levels were assessed at 48 h post calving. Finally, the potential impact of oral capsaicin on calf welfare was investigated by monitoring key physiological parameters.

## 2. Materials and Methods

### 2.1. Animals and Farm

The experiment was conducted from June 2023 to October 2024 on a commercial pasture-based dairy farm located in Cuenca, Ecuador (latitude −3.0576452, longitude −79.0397458), at an altitude of 2640 m above sea level. The farm experiences an average ambient temperature of 11 °C and an annual mean precipitation of 677 mm. It maintains a herd of 64 lactating dairy cows, comprising Holstein × Criollo crossbreeds, with an average milk yield of 16.46 L/cow/day under a semi-extensive grazing system. Cows are artificially inseminated using commercial Holstein semen.

Throughout the study, ethical considerations were observed in accordance with the guidelines outlined in the *Terrestrial Animal Health Code*, Chapter 7.8: “Use of animals in research and education”, issued by the World Organization for Animal Health [45].

To minimise potential biases in TPI associated with dystocia or maternal health disorders, only multiparous cows with a mean body weight of 633.1 ± 10.93 kg, an average parity of 2.7 ± 0.15, an age of 4.8 ± 0.20 years, a body condition score (BCS) of 2.7 ± 0.04 as described by Edmonson et al. [46], and a mean milk yield of 19.6 ± 0.58 L/cow/day during the previous lactation as mothers of the calves were included in the experiment.

### 2.2. Prepartum Management and Neonatal Monitoring

Fifteen days prior to parturition, pregnant cows were transferred to a prepartum paddock. On the day of labour onset, continuous monitoring was carried out until calving was completed and the calf was separated from the dam.

To ensure maximal uniformity, eight pairs of female calves born consecutively from unassisted (eutocic) parturitions were selected. Only calves exhibiting good vitality, with birth weights between 30 and 40 kg and a maximum weight difference of 2.5 kg between paired individuals, were included in the study.

Immediately after birth, each calf remained with the dam under direct supervision by the research team to allow maternal licking and bonding, but preventing him from sucking colostrum from his mother. Calves were then separated and housed individually in separate pens of 2 × 1.2 m^2^, with a cement floor covered with a 5 cc layer of wood shavings.

Postnatal care comprised the following procedures: (1) removal of birth residues; (2) assessment of vitality; (3) measurement of physiological parameters; (4) weight determination; (5) blood sampling; (6) randomization; and (7) colostrum administration.

Each calf was continuously monitored for one hour post-treatment to detect any adverse effects of capsaicin supplementation, and then subsequently monitored for the full 48 h duration of the experiment.

### 2.3. Colostrum Management

To ensure uniformity in colostrum quality, a colostrum bank was established using the first milking of 22 crossbred Holstein cows aged between 3 and 7 years, with parity ranging from 2 to 5 and a prepartum body condition score (BCS) between 3.0 and 3.75 on a 1-to-5 scale. These cows originated from six pasture-based dairy farms located in the region. During the first milking, between 5 and 10 L of colostrum were collected per cow. Refractometer readings ranged from 18 to 26° Brix, resulting in a pooled volume of 158 L, with an average of 21° Brix and an IgG concentration of 50.75 g/L, as determined by ELISA. It was pasteurised at 60 °C for 60 min using a pasteurisation unit (MILKY^®^ FJ 15, Praga, Czech Republic), following the protocol described by Kertz et al. [47].

The pasteurised colostrum was refrigerated for no more than one week [48], then homogenised and stored at −20 °C in 2 L bags until use. This protocol ensured that all calves received colostrum of consistent quality. Prior to administration, colostrum was thawed in a water bath at 37 °C, in accordance with the method described by Jones et al. [49].

### 2.4. Randomization and Feeding Protocol

Each calf was randomly assigned to one of two treatments: control (CON), receiving unsupplemented colostrum, or capsaicin-supplemented (CAP), receiving colostrum supplemented with capsaicin (Capsaicin/Chilli Pepper Extract Power, 99% capsaicin, KAN Phytochemicals Pvt. Ltd., Sonipat, Haryana, India).

**Figure 1:**
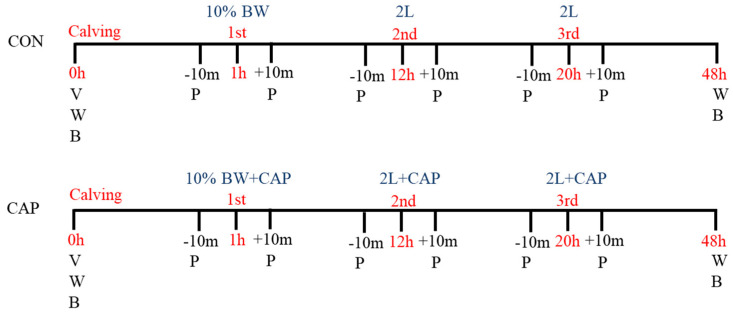
Experimental protocol. CON = Control group; CAP = Group supplemented with 40 mg of capsaicin. V = Vitality assessment (Calf VIGOR Score); W = Body weight measurement; B = Blood sampling; P = Physiological evaluation (heart rate, respiratory rate, and rectal temperature, assessed 10 min before colostrum feeding “-10 m” and 10 min after “+10 m”); 10% BW = First colostrum dose equivalent to 10% of body weight; 2 L = 2 L of colostrum at 12 h (second dose) and at 20 h (third dose).

Within the first hour after calving, all calves received colostrum equivalent to 10% of their body weight via orogastric tubing (Antahi^®^ Trusti Tuber, Wellington, New Zealand) as recommended by Godden et al. [16]. A second dose of 2 L of colostrum was administered 12 h after the first, followed by a third 2 L dose 8 h later. All doses were delivered via oesophageal tubing. From 24 to 48 h of age, all calves received 2 L of commercial milk replacer every 12 h. In the CAP group, colostrum was supplemented with capsaicin at a dosage of 40 mg/kg of body weight for each of the three feedings within the first 24 h. The control group (CON) received only unsupplemented colostrum (see Figure 1).

### 2.5. Clinical Evaluation

Body weight was measured at 1 h and 48 h of age using a digital scale (EziWeigh7i; Tru-Test Livestock Management, County Cork, Ireland). Heart rate (HR) and respiratory rate (RR) were determined by auscultation, and rectal temperature was measured using a digital thermometer. All physiological parameters were assessed 10 min before and 10 min after each colostrum administration (at 1, 12, and 20 h).

Vitality at birth was evaluated using the Calf VIGOR Score [50]. which included visual assessment of the following: (1) evidence of meconium staining and general appearance of the tongue and head; (2) initiation of movement and time to achieve postural reflexes; (3) responsiveness to stimuli (e.g., nasal mucosa stimulation with a straw, tongue pinch, ocular touch); (4) oxygenation status based on mucosal membrane colour and tongue protrusion; (5) physiological parameters, including HR and RR. Vitality scores were classified as follows: 0–1 = excellent vitality; 2–4 = very good vitality; 5–6 = good vitality; 7–10 = marginal vitality; ≥11 = poor vitality. Only calves with excellent vitality were included in the study.

### 2.6. Sampling and Laboratory Analyses

The IgG concentration in the pooled colostrum was determined using a competitive ELISA test for IgG quantification in colostrum (MonoScreen QuantELISA Inmunoglobulin Easy BIO K 420, Bio-X Diagnostics, Rochefort, Belgium).

To assess plasma concentrations of IgG, total protein (TP), albumin (Alb), metabolites, and enzymatic profiles, blood samples were collected by jugular venipuncture at two time points: at 1 h of age (prior to first colostrum intake) and at 48 h post calving. Blood was collected into 5 mL Vacutainer^®^ serum tubes, stored in coolers, and transported to the laboratory. Samples were centrifuged at 2500 rpm for 15 min, and serum was transferred into Eppendorf tubes and stored at −20 °C until analysis.

IgG concentration was measured using a competitive ELISA for bovine serum immunoglobulins (MonoScreen QuantELISA Inmunoglobulina Easy BIO K 420, Bio-X Diagnostics, Rochefort, Belgium).

Serum concentrations of TP, Alb, glucose, blood urea nitrogen (BUN), cholesterol, triglycerides, alkaline phosphatase (ALP), aspartate aminotransferase (AST), alanine aminotransferase (ALT), and lactate dehydrogenase (LDH) were measured by spectrophotometry (Mindray^®^ BS-240Pro, Henzhen Mindray Bio-Medical Electronics Co., Ltd., Shenzhen, China).

### 2.7. Statistical Analysis

Data were analysed using SPSS software version 29 (IBM, Armonk, NY, EUA) and results were considered statistically significant at *p* < 0.05. Normality was assessed using the Shapiro–Wilk test. Non-normally distributed data were log10-transformed.

Student’s *t*-test was used to compare IgG, TP, and Alb concentrations between the CON and CAP groups. Analysis of metabolites and enzymatic profiles was performed using ANOVA, followed by Tukey’s post hoc test at the 5% significance level. Pearson’s correlation was used to assess the relationship between mean population values and IgG concentrations at 1 h and 48 h. Results are presented as mean ± standard deviation in tables, and as mean ± standard error in figures.

## 3. Results

### 3.1. Calf Weight

The mean calving weight (±SD) of the calves (*n* = 16) was 36.5 ± 0.49 kg. When grouped, the control group (CON) had a mean weight of 36.7 ± 1.10 kg, while the capsaicin group (CAP) averaged 36.2 ± 2.64 kg, with no significant difference (*p* = 0.961). At 48 h of age, the mean weight was 38.5 ± 1.11 kg in the CON group and 38.0 ± 2.82 kg in the CAP group, again with no significant difference (*p* = 0.966).

### 3.2. Physiological Parameters

The mean values of the physiological constants of the study population taken at hours 1, 12, and 20 are detailed in Table 1. RH increases as the hours pass. Conversely, RR decreases as the hours pass and then stabilises. Temperature increases until 12 noon and then remains stable.

Capsaicin administration in the first colostrum feeding (1 h) led to a significant increase in HR measured 10 min after ingestion, compared to values recorded 10 min before ingestion (*p* < 0.05). In contrast, HR remained unchanged in the CON group (*p* > 0.05; Table 2). No significant changes in RR or RT were observed before or after the first colostrum feeding in either group (*p* > 0.05).

Similarly, no significant differences were observed in HR, RR, or RT before and after the second feeding (12 h; Table 3).

In the third feeding at 20 h, physiological parameters remained consistent within each group (*p* > 0.05; Table 4).

### 3.3. Calf Vitality Score

Calf vitality, assessed using the Calf VIGOR Score, showed a population mean of 1.2 ± 0.20, indicating excellent vitality (score 0–1) and fulfilling one of the inclusion criteria. The CON group had a mean score of 1.3 ± 0.36, while the CAP group scored 1.1 ± 0.22, with no significant difference between groups (*p* > 0.05).

### 3.4. Population Concentration of IgG, PT, and Albumin

At 48 h, IgG concentration increased 24.3-fold compared to values at 1 h of age (*p* < 0.0001). TP increased by 64% (*p* < 0.0001), while Alb showed a non-significant 3% increase compared to baseline values (*p* = 0.370; Table 5).

### 3.5. Serum of IgG, PT, and Alb by Experimental Groups

**Figure 2:**
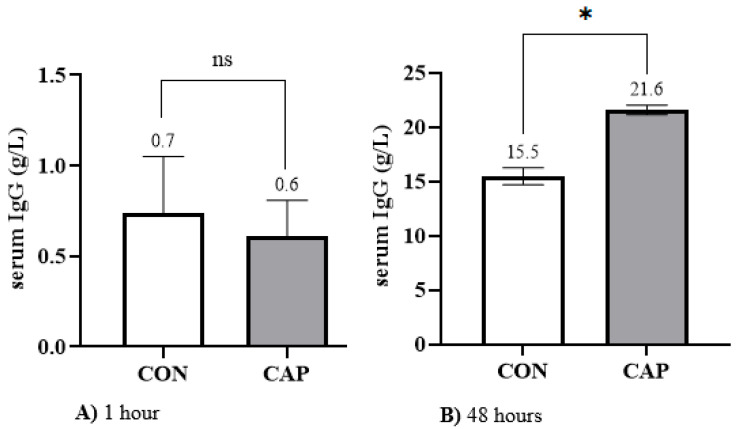
Mean ± standard error of serum immunoglobulin G (IgG) concentration measured at 1 h ((**A**): 1 h) and 48 h ((**B**): 48 h) after calving in control (CON) and capsaicin-supplemented (CAP) groups. Student’s *t*-test, *p* < 0.05. * Indicates statistically significant differences between groups. ns: no difference between groups.

At 1 h of life, serum IgG concentrations were similar between groups (CAP: 0.6 ± 0.19 g/L vs. CON: 0.7 ± 0.31 g/L; *p* > 0.05; Figure 2A). However, at 48 h, calves in the CAP group exhibited significantly higher IgG concentrations (21.6 ± 0.43 g/L) compared to the CON group (15.5 ± 0.78 g/L; *p* < 0.05; Figure 2B).

**Figure 3:**
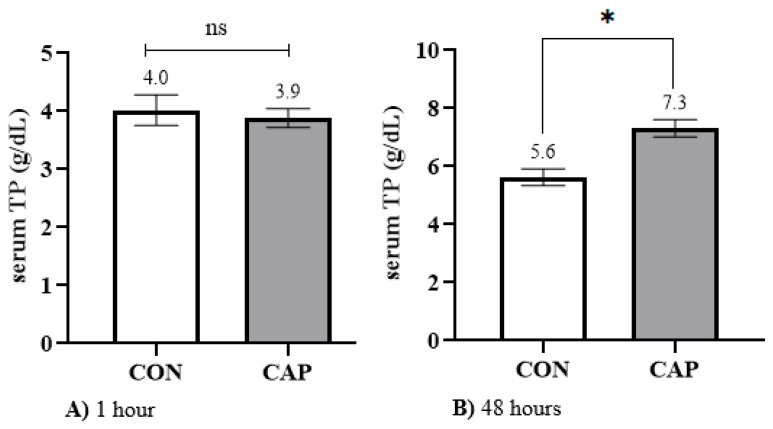
Mean ± standard error of serum total protein (TP) concentration measured at 1 h ((**A**): 1 h) and 48 h ((**B**): 48 h) after calving in control (CON) and capsaicin-supplemented (CAP) groups. Student’s *t*-test, *p* < 0.05. * Indicates statistically significant differences between groups. ns: no difference between groups.

At 1 h of age, serum TP concentrations were comparable between the CAP (3.9 ± 0.16 g/dL) and CON (4.0 ± 0.26 g/dL) groups (*p* > 0.05; Figure 3A). However, by 48 h, the CAP group exhibited significantly higher TP levels (7.3 ± 0.29 g/dL) compared with the CON group (5.6 ± 0.28 g/dL; *p* < 0.05; Figure 3B).

**Figure 4:**
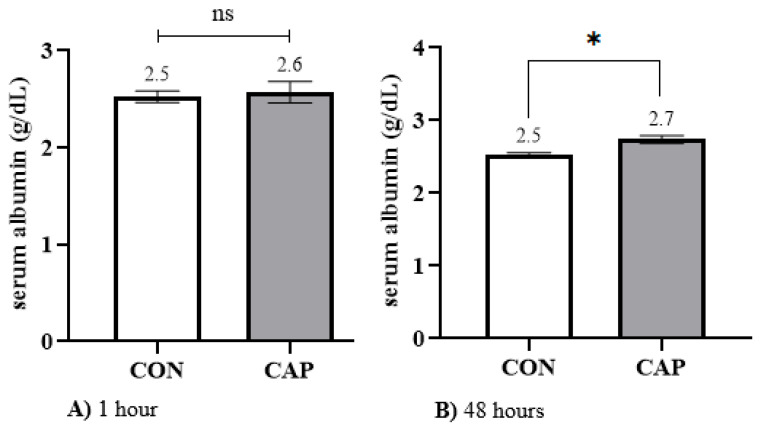
Mean ± standard error of serum albumin (Alb) concentration measured at 1 h ((**A**): 1 h) and 48 h ((**B**): 48 h) after calving in control (CON) and capsaicin-supplemented (CAP) groups. Student’s *t*-test, *p* < 0.05. * Indicates statistically significant differences between groups. ns: no difference between groups.

Albumin concentrations were similar between groups at 1 h of age (CAP: 2.6 ± 0.10 g/dL vs. CON: 2.5 ± 0.05 g/dL; *p* > 0.05; Figure 4A). However, at 48 h, albumin levels were significantly higher in the CAP group (2.7 ± 0.05 g/dL) compared with the CON group (2.5 ± 0.04 g/dL; *p* < 0.05; Figure 4B).

### 3.6. Correlation Between IgG, PT, and Albumin

Pearson correlation analysis showed no significant associations between IgG at 1 h and either TP or Alb at 1 or 48 h (Table 6). However, IgG at 48 h was moderately and significantly correlated with TP at 48 h (*r* = 0.65; *p* < 0.001). No correlation was observed between IgG at 48 h and Alb, or between TP at 1 h and the other parameters (Table 6).

### 3.7. Metabolite Results

At 1 h of age, blood glucose levels were similar between CAP (84.2 mg/dL) and CON (82.0 mg/dL) groups (Table 7). In the CON group, glucose levels remained stable at 48 h (83.5 mg/dL; *p* > 0.05). In contrast, the CAP group exhibited a significant increase of 37.5 mg/dL, reaching 117.7 mg/dL at 48 h (*p* < 0.05) compared to both its own 1 h value and the values recorded in the CON group at 1 and 48 h.

Blood urea nitrogen (BUN) concentrations at 1 h were similar between the CAP (10.9 mg/dL) and CON (12.3 mg/dL) groups (*p* > 0.05) and remained stable at 48 h (CON: 12.4 mg/dL vs. CAP: 11.8 mg/dL; *p* > 0.05). Cholesterol levels at 1 h were also comparable (CAP: 17.3 mg/dL vs. CON: 17.5 mg/dL; *p* > 0.05) but increased significantly at 48 h to 17.9 mg/dL in the CON group and 34.6 mg/dL in the CAP group (*p* < 0.05). The 48 h value in the CAP group was 1.5 times higher than that of the CON group (*p* < 0.05). Triglyceride concentrations at 1 h were 4.5 mg/dL in the CON group and 4.9 mg/dL in the CAP group (*p* > 0.05) but rose significantly by 48 h to 33.2 mg/dL and 57.0 mg/dL, respectively. This represented a 1.6-fold increase in the CAP group compared to the CON group at 48 h (*p* < 0.05).

### 3.8. Enzymatic Profile Values

At 1 h of life, ALP activity ranged from 196.54 to 293.5 U/L in the CON group and from 228.92 to 373.77 U/L in the CAP group, with no significant difference between groups (*p* > 0.05). However, at 48 h, ALP levels in the CAP group increased significantly to 431.61–618.01 U/L, compared to 171.14–366.41 U/L in the CON group (*p* < 0.05; Table 8).

At 1 h of life, AST activity was similar between groups (CAP: 19.2 U/L vs. CON: 18.0 U/L; *p* > 0.05). At 48 h, AST levels increased significantly in both groups (CAP: +46.7 U/L; CON: +35.2 U/L; *p* < 0.05), with the CAP group exhibiting a 1.2-fold greater increase compared to the CON group (*p* < 0.05).

ALT levels at 1 h were 5.1 U/L in the CON group and 4.9 U/L in the CAP group, with no significant difference between groups (*p* > 0.05). At 48 h, both groups exhibited significant increases (CAP: 17.8 U/L; CON: 13.5 U/L; *p* < 0.05), but the overall trend was similar. Lactate dehydrogenase (LDH) The CON group as in the CAP group remained stable at 48 h decreased in the CON group and increased in the CAP group, but the overall trend was similar across both groups.

## 4. Discussion

The TPI depends on various factors related to both the colostrum and the calf. Regarding colostrum, the most relevant aspects include its quality, volume administered, timing, and method of administration [51]. In relation to the calf, the most critical factor is its vitality at calving, which is directly influenced by the ease of parturition, as maternal–foetal disproportion often results in dystocia and the calving of acidotic calves [52].

In this experiment, we exclusively used multiparous cows, which are known to have a lower incidence of dystocia compared to primiparous heifers [53]. Furthermore, male calves have been reported to experience higher rates of dystocia than females [54], hence, only female calves were included in the study.

Calving weight is another important factor associated with the risk of dystocia, which may compromise neonatal vitality and increase the likelihood of FTIP. Therefore, only female calves with birth weights ranging from 30 to 40 kg were selected, with differences between the control (CON) and capsaicin (CAP) groups kept below 2.5 kg. The average calving weight was 36.5 ± 0.49 kg, consistent with that reported for Holstein heifer calves (36.2 kg; [55]). However, other studies have described higher calving weights (46.5 kg) in calves born to multiparous Holstein dams [56].

To minimise variability in colostrum quality, a colostrum bank was established using material from cows of the same breed (crossbred Holstein × Criollo). Selection criteria for donor animals included age, parity, body condition score (BCS), and average colostrum yield at first milking, as breed has been reported to influence colostrum quality [57].

An important factor to consider is the altitudinal zone at which the study was conducted (2500 masl). This area has its own environmental conditions, which have been described as influencing physiological constants such as HR, RR, RT, and feeding [44]. In these locations, the body compensates for the lower oxygen availability (hypobaric hypoxia) by increasing HR to deliver more oxygen to the tissues and, to do so, increasing pulmonary ventilation (RR), which leads to a decrease in appetite. Furthermore, neonatal hypoxia has been shown to affect IgG absorption by delaying intestinal closure for up to 40.5 h, as long as the calf has ingested colostrum at birth [58].

### 4.1. Effect of Capsaicin on Physiological Parameters

The effect of capsaicin on physiological parameters is of intrinsic interest and serves as a means to assess potential adverse impacts on animal welfare.

When the physiological data were compared between the two experimental groups (CON and CAP) across the three colostrum feedings (1, 12, and 20 h), and at two different time points (10 min before and 10 min after each colostrum intake), it was observed that the calves receiving capsaicin in the first colostrum feeding (1 h) exhibited a heart rate (HR) that was 12.4 bpm higher than that of the CON group (*p* < 0.05; Table 2).

This increase in HR in calves that were administered capsaicin is likely attributable to the action of capsaicinoids, which are known to exert irritant effects and may initially induce discomfort and pain upon ingestion [43]. Such irritation is perceived by the organism as a stressor, potentially activating the hypothalamic–pituitary–adrenal (HPA) axis and leading to the release of cortisol. Among its physiological effects, cortisol is known to cause a transient increase in HR [42].

However, it has been reported that repeated and prolonged exposure to capsaicin induces desensitisation of the TRPV1 receptor, thereby preventing nociceptive stimuli from triggering the release of inflammatory neuropeptides into the synaptic cleft. As a result, the transmission of painful stimuli and the perception of pain at the spinal cord level are inhibited [43].

This desensitisation was reflected in the second heart rate (HR) assessment following colostrum intake at 12 h (Table 3)**,** where calves in the capsaicin (CAP) group showed a reduction of 4.4 bpm compared to the HR recorded at 1 h. However, in the third assessment, conducted 10 min after the final colostrum feeding, the HR in the capsaicin-treated group appeared to stabilise at 143.6 ± 8.44 bpm (Table 4).

The results obtained in this experiment confirm, on the one hand, a fundamental pharmacodynamics property of capsaicin—its initial irritant effect followed by subsequent desensitisation—and, on the other hand, are consistent with the findings of Santos et al. [59], who reported a slight decrease in HR from the first to the second day of life, with values approaching 140 bpm. Nonetheless, this trend was not observed in respiratory rate (RR) and rectal temperature (RT), which did not differ significantly between the experimental groups (*p* > 0.05).

Conversely, RR exhibited a decline of 7.6 breaths per minute (rpm) at 12 h, reaching a stable value of 47 rpm in the assessment performed at 20 h post-colostrum administration (Table 3 and Table 4). This pattern mirrors the decrease and subsequent stabilisation in RR, as described by Santos et al. [59], in Holstein calves raised under intensive production systems. However, it is important to note that the RR values recorded in our study were lower than those reported by the authors (ranging from 60 to 55 rpm).

RT values were comparable (*p* > 0.05) between the two time points of evaluation (10 min before and 10 min after colostrum intake) across the first, second, and third colostrum feedings (Table 2, Table 3 and Table 4), indirectly indicating that the capsaicin dose used did not cause significant irritation in the animals—a factor that could have otherwise contributed to post-administration hyperthermia.

When assessing the RT curve over the first, second, and third colostrum feedings, both experimental groups displayed similar values, with a slight increase in temperature (0.6 °C in the CON group and 0.8 °C in the CAP group), which was not statistically significant (*p* > 0.05; Table 2, Table 3 and Table 4).

This RT pattern may be linked to one of the physiological roles of capsaicin—thermoregulation—primarily mediated through activation of the transient receptor potential vanilloid 1 (TRPV1) receptor. This activation depends on various factors, including the time elapsed since administration, the dose, and the frequency of application, and may result in either immediate or delayed effects. In a study conducted by Szolcsányi [60], the administration of a single, minimal dose of capsaicin induced mild hypothermia, attributed to the release of pro-inflammatory peptides. Conversely, when capsaicin is administered regularly over an extended period, TRPV1 receptor desensitisation occurs, leading to increased RT [61]. Based on this mechanism, it is reasonable that no increase in RT was observed 10 min after colostrum administration with capsaicin, given the low dose and the fact that it was administered at only three discrete time points.

However, the RT recorded in our calves at 1 h of life was 37.6 ± 0.30 °C, which is approximately 1.4 °C below the optimal temperature range reported for neonatal calves (39.0–39.5 °C; [62]). This deviation may be attributed to high-altitude conditions (2640 m above sea level) and environmental factors at the dairy farm where the calves were born. Notably, over 70% of parturitions occurred at night in open maternity paddocks, where ambient temperatures ranged from 13 to 20 °C between day and night, with a perceived temperature of 8 to 12 °C. These rearing conditions differ substantially from the controlled-environment intensive systems used in the study by Szenci et al. [62].

To the best of our knowledge, no published data are currently available regarding physiological parameters within the first hour of life in crossbred Holstein × Criollo heifer calves. Consequently, the values reported herein may serve as useful reference points for future research in this genotype. Based on the aforementioned findings, we conclude that the administration of capsaicin via an orogastric tube in conjunction with colostrum does not appear to adversely affect calf welfare.

### 4.2. Effect of Capsaicin on TIP (IgG, TP, Albumin)

#### 4.2.1. IgG

In this study, the mean population IgG concentration at 1 h of life was found to be low (0.7 ± 0.17 g/L). Similarly, the values recorded in the CAP (0.6 ± 0.19 g/L) and CON (0.7 ± 0.31 g/L) groups were comparable. These low concentrations at birth are consistent with the limited passive immunity transferred from dam to offspring via the bovine cotyledonary synepitheliochorial placenta [63,64].

For this reason, all calves in the study received colostrum via an oesophageal tube on three occasions within the first 24 h of life. The colostrum was sourced from a single pool with a confirmed IgG concentration of 50.75 g/L. This protocol enabled the study population to reach a mean IgG concentration of 15.5 ± 0.78 g/L at 48 h of age, placing them in the “Fair” category, as defined by the consensus report of Godden et al. [7].

The rapid increase in IgG levels during this period is directly linked to the capacity of the neonatal small intestine to absorb high-molecular-weight proteins and other macromolecules present in colostrum through non-selective uptake [65]. This absorptive ability persists only for a limited time and is mediated via pinocytosis, supported by the presence of immature enterocytes, and characterised by abundant vacuoles and an enhanced capacity for macromolecular uptake [66].

Notably, when 40 mg of capsaicin per kg of body weight was added to the colostrum administered to the CAP group, a 39.35% increase in IgG concentration was observed compared to the CON group (Figure 2), reaching a mean of 21.6 ± 0.43 g/L. This value falls within the “Good” category, according to the thresholds proposed by Godden et al. [7], and exceeds the minimum standard for adequate passive transfer. We attribute this improvement in TPI to the supplementation of capsaicin in the CAP group. To the best of our knowledge, this is the first study to evaluate the addition of capsaicin to colostrum with the specific aim of enhancing TPI in neonatal calves during the first 48 h of life.

Nevertheless, previous studies have shown that capsaicin significantly affects the expression of transporter proteins, particularly those involved in drug metabolism and the regulation of the transcellular pathway through enterocytes, which may enhance the non-selective absorption of macromolecules [37].

Moreover, capsaicin is known to be efficiently and passively absorbed in the stomach and upper small intestine [67], where it activates TRPV1 channels in the gastrointestinal epithelium. This activation triggers a range of physiological responses via stimulation of intestinal mucosal afferent nerves, including increased local blood flow [68].

It has also been demonstrated that capsaicin intake induces the releases of calcitonin gene-related peptide (CGRP), which activates cyclooxygenase-1 (COX-1), an enzyme with gastroprotective properties. This activation subsequently enhances the absorptive surface of the small intestine by elongating and thickening the microvilli, and by altering membrane permeability, thereby facilitating the absorption of minerals such as zinc. However, this mechanism was not directly evaluated in the present study, highlighting the need for a second experimental phase aimed at histological analysis of the intestinal microvilli in animals supplemented with capsaicin [69].

Based on the above, it is plausible that these properties of capsaicin contributed to the enhanced absorption of IgG and, consequently, the improved TPI observed in this experiment.

#### 4.2.2. TP

The present study also demonstrated that the total protein (TP) concentration in the study population increased by 40% at 48 h of life compared to the value recorded at 1 h (Table 5). This increase is consistent with the quality and volume of colostrum administered, as well as the timing of its delivery [70,71,72]. A mean TP concentration of 5.6 ± 0.28 g/dL was reached, placing the value within the “Fair” category, as defined by Godden et al. [7].

Furthermore, the concentration determined in this study aligns with that reported by Deelen [73], who observed a mean TP level of 6 ± 0.8 g/dL, with a reference range of 4.4 to 8.8 g/dL, which is considered indicative of good passive immunity transfer. These findings confirm that the TP values observed in the population were within normal physiological ranges when calves were supplied with colostrum of adequate quality, as assessed by a Brix value of 21%.

However, when capsaicin was administered alongside colostrum, a 30.4% increase in TP concentration at 48 h was observed relative to the population mean, reaching 7.3 ± 0.29 g/dL. This value corresponds to the “Excellent” category—two levels above that of the control group. These results indicate that intestinal stimulation by capsaicin may enhance the absorption of total proteins, potentially exceeding the effect observed on IgG uptake. As previously discussed, this phenomenon could be associated with an increase in the absorptive surface area of the intestine—a mechanism well documented in avian species [74], although data in bovines remain scarce. The increase in TP concentration observed at 48 h showed a strong positive correlation with IgG concentration at the same time point (r = 0.65; *p* < 0.001), further supporting the validity of our findings (Table 6).

#### 4.2.3. Albumin

When evaluating Alb concentration at 1 h versus 48 h of life in the study population, no significant variation was observed (*p* = 0.37), with mean values remaining stable at 2.5 g/dL. It should be noted that albumin accounts for approximately 35 to 50% of total plasma protein (TP), yet its concentration remains relatively stable in neonatal calves, typically ranging from 2.5 to 3 g/dL [75]. This low concentration is directly related to the physiological principle whereby albumin and IgG are the main proteins contributing to plasma oncotic pressure. Consequently, a simultaneous increase in the concentrations of both proteins would elevate osmotic pressure; therefore, it is essential that albumin levels remain low to compensate for the rise in IgG, thereby maintaining osmotic equilibrium in the bloodstream [76].

The addition of capsaicin during the colostrum feeding process did not result in any change in serum albumin concentration (2.5 g/dL), unlike the variations observed in IgG and TP levels. This finding may be attributed, on one hand, to the haemodilution occurring after the calf’s initial feeding [77], and on the other, to the compensatory physiological mechanisms aimed at maintaining osmotic homeostasis [76]. This characteristic is further reflected in the low and non-significant correlation observed between IgG and albumin concentrations (r = 0.48; *p* = 0.06).

### 4.3. Metabolites

#### 4.3.1. Glucose

The results showed that glucose values prior to 1 h of life were low, generally ranging between 54.0 and 139.8 mg/dL. This initial hypoglycaemia is physiologically associated with the fact that, before calving, the neonate meets its glucose requirements via placental transfer from dam to foetus [78]. The values observed in our study are consistent with those reported for Holstein calves at 24 h of age kept under confined systems in tropical lowland conditions (68 mg/dL; [59]).

However, after calving, glucose requirements are met by the ingestion of colostrum and milk, which provide lactose and fat as the primary energy sources [79]. Therefore, although calves are born with low blood glucose levels, these rise rapidly following lactose absorption from colostrum [80], contributing to the maturation of the calf’s gluconeogenic capacity [81]. Furthermore, this postnatal increase in glucose is enhanced by elevated corticosteroid concentrations after parturition [76]. At 48 h, the CON group maintained low glucose levels (83.5 ± 19.98 mg/dL), whereas the CAP group showed a significant increase to 117.7 ± 16.94 mg/dL. This rapid rise in glucose observed in the CAP group is comparable to the findings of Egli and Blum [77], who also reported lower glucose concentrations on day 1, followed by an increase on day 2.

#### 4.3.2. BUN

Blood urea nitrogen concentrations were low in both the CON and CAP groups, both at calving (12.3 ± 0.22 and 10.9 ± 0.91 mg/dL, respectively) and at 48 h (12.3 ± 0.94 and 11.8 ± 0.54 mg/dL, respectively). This reduced serum concentration after colostrum intake may be attributed to the presence of non-nutritional factors in colostrum, such as IGF-I and insulin, which exert anabolic effects on protein metabolism, thereby reducing plasma urea levels [82]. It is also important to consider that urea concentration in neonates—provided there is no hepatic or renal dysfunction—depends on absorption (from colostrum), as well as protein synthesis and degradation [83]. Nevertheless, the values obtained in this study fall within the normal reference ranges for neonatal calves [84]. 

#### 4.3.3. Cholesterol

The neonate’s rapid ability to adapt to a postnatal energy source shift—from primarily carbohydrate-based during foetal life to one rich in fats and relatively low in carbohydrates provided by colostrum [7]—enables the first colostrum intake to significantly contribute to the development of the lipid profile [80].

In our study, cholesterol concentrations at calving were low in both groups: 17.5 ± 2.55 mg/dL in the CON group and 17.3 ± 1.74 mg/dL in the CAP group. By 48 h, an increase was observed in both groups; however, the rise was significantly greater in the CAP group (51.9 ± 7.66 mg/dL) compared to the CON group (35.4 ± 2.39 mg/dL). The 1.7-fold increase in cholesterol observed in the CAP group relative to CON may be attributed to the effect of capsaicin on enhancing intestinal villi development, potentially facilitating more efficient lipid absorption from colostrum—an effect previously described for capsaicin.

#### 4.3.4. Triglycerides

The increase in plasma triglyceride concentrations observed in the experimental groups is likely due to high fat intake via colostrum [85]. It is also important to note that lipid levels in neonatal blood plasma depend on both the quantity and timing of colostrum administration [80]. Once again, the increase observed at 48 h showed differences between groups, with the CAP group exhibiting values 1.6 times higher than the CON group, indicating a potential modulatory effect of capsaicin supplementation on lipid metabolism.

### 4.4. Enzyme Profile

AST and ALT concentrations prior to 1 h of life were low, which may be attributed to the inherently reduced enzymatic activity in neonatal calves. However, a transient increase was observed after calving, likely due to the absorption of these enzymes from ingested colostrum [86,87]. 

Therefore, the statistically significant differences in the concentrations of both enzymes at 48 h can be attributed to their absorption from colostrum, with the increase being significantly greater in the case of AST—an effect that may be influenced by capsaicin. A similar pattern was observed for alkaline phosphatase, where the differences between the CAP and CON groups at 48 h could be attributed to capsaicin’s effect on intestinal absorption, as previously discussed.

Egli and Blum [77] suggested that the transient rise in plasma AST and ALP concentrations during the first 24 h of life results from enzyme absorption via colostrum, which is consistent with the findings of our study. This is further supported by Kurtz and Willett [88] and Hammon and Blum [89], who reported increased AST activity at 24 and 48 h postpartum. Although AST plays a key role in gluconeogenesis, neonatal calves—being functionally monogastric—do not rely on this metabolic pathway to maintain glycaemia, but rather on intestinal absorption [90]. Nevertheless, Zanker et al. [91] proposed that the postnatal increase in AST is not necessarily associated with the timing of first colostrum ingestion, and may instead be of endogenous origin, independent of colostrum consumption.

Regarding ALP, the increase observed at 48 h aligns with the findings of Fay [92], who noted a potential association with colostral absorption. However, it is also plausible that the increase reflects greater endogenous synthesis, independent of colostrum intake.

Kurz [88] reported a gradual increase in AST and ALT during the first 24 h of life, a pattern consistent with the findings of our study. The author also noted that such increases represent a physiological response to colostrum intake, influenced by the age of the calf and the timing of the first feeding. Thus, enzymatic activity in neonates may increase due to either intestinal absorption from colostrum—potentially stimulated by capsaicin—or endogenous production.

## 5. Conclusions

Under the conditions of our study, capsaicin supplementation in colostrum improved serum IgG concentrations in calves at 48 h of age. Furthermore, this addition improved the availability of glucose, cholesterol, and triglycerides. This supplementation of capsaicin was associated with increased concentrations of alkaline phosphatase, aspartate aminotransferase, and alanine aminotransferase, suggesting improved health status in calves. A secondary effect of supplementation was an initial increase in HR, possibly a physiological response to the irritant effect of capsaicin; however, this increase was not sustained during the second or third colostrum feedings, indicating that capsaicin can be safely administered in successive doses. Capsaicin also had no significant effects on RF or RT at any of the three colostrum administration times. This suggests that no significant or lasting changes occurred in the calves’ physiological parameters, supporting the safety of capsaicin as a colostrum additive at this altitude. However, we believe that further studies are needed to confirm the results. In summary, the use of capsaicin as a colostrum additive represents a promising strategy to enhance plasma immunoglobulin transfer in Holstein-crossbred heifer calves without inducing significant adverse effects on their physiological parameters.

## Figures and Tables

**Table 1:** Mean ± standard deviation of heart rate (HR), respiratory rate (RR), and rectal temperature assessed at 1 h, 12 h, and 20 h of life of the calf population.

	1 h	12 h	20 h
Heart rate (HR)	139.1 ± 6.92	140.9 ± 7.29	142.0 ± 7.69
Respiratory rate (RR)	48.8 ± 7.19	43.9 ± 4.44	45.1 ± 6.55
Rectal temperature (°C)	37.6 ± 0.30	38.0 ± 0.37	38.1 ± 0.29

Calf assessment time: 1 h; first hour. 12 h; 12 h. 20 h; 20 h after birth.

**Table 2:** Mean ± standard deviation of heart rate (HR), respiratory rate (RR), and rectal temperature measured 10 min before and 10 min after administration of the first colostrum dose (1 h of life) in control (CON) and capsaicin-supplemented (CAP) groups.

	CON		CAP	
	Before	After	Before	After
Heart rate (HR)	136.8 ± 2.38 A	137.4 ± 1.51 A	134.8 ± 3.27 a	147.2 ± 9.67 b
Respiratory rate (RR)	46.8 ± 9.12 A	48.4 ± 9.68 A	45.8 ± 3.83 a	54.2 ± 0.83 a
Rectal temperature (°C)	37.7 ± 0.40 A	37.5 ± 0.37 A	37.5 ± 0.20 a	37.8 ± 0.16 a

Different uppercase letters (A) within a row indicate statistically significant differences between pre- and post-administration values in the control group (CON). Different lowercase letters (a, b) within a row indicate statistically significant differences between pre- and post-administration values in the capsaicin group (CAP). Tukey’s post hoc test, *p* < 0.05.

**Table 3:** Mean ± standard deviation of heart rate (HR), respiratory rate (RR), and rectal temperature measured 10 min before and 10 min after administration of the second colostrum dose (12 h of life) in control (CON) and capsaicin-supplemented (CAP) groups.

	CON		CAP	
	Before	After	Before	After
Heart rate (HR)	141.0 ± 8.66 A	143.2 ± 6.09 A	136.6 ± 7.40 a	142.8 ± 7.15 a
Respiratory rate (RR)	41.2 ± 1.78 A	45.6 ± 5.12 A	42.2 ± 2.48 a	46.6 ± 5.68 a
Rectal temperature (°C)	37.9 ± 0.38 A	38.0 ± 0.53 A	37.9 ± 0.26 a	38.3 ± 0.04 a

Different letters uppercase (A) within a row indicate statistically significant differences between pre- and post-administration values in the control group (CON). Different lowercase letters (a) within a row indicate statistically significant differences between pre- and post-administration values in the capsaicin group (CAP). Tukey’s post hoc test, *p* < 0.05.

**Table 4:** Mean ± standard deviation of heart rate (HR), respiratory rate (RR), and rectal temperature measured 10 min before and 10 min after administration of the third colostrum dose (20 h of life) in control (CON) and capsaicin-supplemented (CAP) groups.

	CON		CAP	
	Before	After	Before	After
Heart rate (HR)	139.4 ± 5.72 A	146.0 ± 6.67 A	138.8 ± 9.31 a	143.6 ± 8.44 a
Respiratory rate (RR)	44.2 ± 7.69 A	46.0 ± 9.02 A	43.2 ± 3.27 a	47.0 ± 6.44 a
Rectal temperature (°C)	38.0 ± 0.20 A	38.1 ± 0.40 A	37.9 ± 0.16 a	38.3 ± 0.13 a

Different uppercase letters (A) within a row indicate statistically significant differences between pre- and post-administration values in the control group (CON). Different lowercase letters (a) within a row indicate statistically significant differences between pre- and post-administration values in the capsaicin group (CAP). Tukey’s post hoc test, *p* < 0.05.

**Table 5:** Mean ± standard deviation of serum immunoglobulin G (IgG), total protein (TP), and albumin (Alb) concentrations measured at 1 h and 48 h after calving in control (CON) and capsaicin-supplemented (CAP) groups.

	*n*	Mean ± SD	Minimum	Maximum	*p*-Value
IgG 1 h (g/L)	16	0.7 ± 0.64	0.01	2.69	0.0001
IgG 48 h (g/L)	8	15.5 ± 2.24	10.27	17.29	
TP 1 h (g/dL)	16	4.0 ± 0.60	3.28	5.62	0.0001
TP 48 h (g/dL)	8	5.6 ± 0.80	4.25	6.56	
Alb 1 h (g/dL)	16	2.6 ± 0.24	2.03	2.95	0.3700
Alb 48 h (g/dL)	8	2.5 ± 0.10	2.37	2.66	

*p*-value: significance values are indicated. *n*: number of calves. SD: standard deviation. g/L: grams/litre. IgG: Immunoglobulin G. TP: Total protein. Alb: Albumin.

**Table 6:** Estimation of Pearson’s correlation between serum immunoglobulin G (IgG), total protein (TP), and albumin (Alb) concentrations measured at 1 h and 48 h after calving in control (CON) and capsaicin-supplemented (CAP) groups.

	PT 1 h		PT 48 h		Alb 1 h		Alb 48 h	
	*r*	*p*	*r*	*p*	*r*	*p*	*r*	*p*
IgG 1 h	0.29	0.27	−0.04	0.88	0.45	0.08	−0.13	0.64
IgG 48 h	0.24	0.93	0.65	0.00	0.07	0.79	0.48	0.06

*r*: Pearson’s correlation; *n*: sample size; *p*: significance values are indicated. PT: Total protein. Alb: Albumin. IgG 1 h: Immunoglobulins G at 1 h. IgG 48 h: Immunoglobulins G at 48 h.

**Table 7:** Mean ± standard deviation of metabolites measured at 1 h (1 h) and 48 h (48 h) after calving in control (CON) and capsaicin-supplemented (CAP) groups.

	CON		CAP	
	1 h	48 h	1 h	48 h
Glucose (mg/dL)	84.2 ± 20.18 a	83.5 ± 19.98 a	82.0 ± 15.44 a	117.7 ± 16.94 b
BUN (mg/dL)	12.3 ± 0.22 a	12.3 ± 0.94 a	10.9 ± 0.91 a	11.8 ± 0.54 a
Cholesterol (mg/dL)	17.5 ± 2.55 a	35.4 ± 2.39 b	17.3 ± 1.74 a	51.9 ± 7.66 c
Triglycerides (mg/dL)	4.5 ± 0.62 a	37.7 ± 3.17 b	4.9 ± 2.17 a	61.9 ± 8.21 c

Blood urea nitrogen (BUN) mg/dL. Student’s *t*-test, *p* < 0.05. Different lowercase letters (a, b and c) within a same line indicate statistically significant differences between groups.

**Table 8:** Mean ± standard deviation of enzymatic profile values measured at 1 h and 48 h after calving in control (CON) and capsaicin-supplemented (CAP) groups.

	CON		CAP	
	1 h	48 h	1 h	48 h
ALP (U/L)	252.9 ± 44.66 a	288.7 ± 90.09 a	328.9 ± 67.49 a	513.3 ± 86.35 b
AST (U/L)	18.0 ± 1.92 a	53.2 ± 3.18 a	19.2 ± 1.57 a	65.9 ± 1.50 b
ALT (U/L)	5.1 ± 1.39 a	13.5 ± 3.48 b	4.9 ± 1.09 a	17.8 ± 6.80 b
LDH (U/L)	1008.3 ± 219.92 a	995.1 ± 101.41 a	1289.6 ± 134.66 a	1315.4 ± 162.34 a

ALP: Alkaline phosphatase; AST: Aspartate aminotransferase; ALT: Alanine aminotransferase; LDH: Lactate dehydrogenase; U/L: International units per litre. Student’s *t*-test, *p* < 0.05. Different lowercase letters (a, b) within a same line indicate statistically significant differences between groups.

## Data Availability

The data that support the findings of this study are available from the corresponding author upon reasonable request.
